# Determining PSMA-617 Mass and Molar Activity in Pluvicto Doses

**DOI:** 10.2967/jnumed.124.269111

**Published:** 2025-05

**Authors:** Ivan E. Wang, Allen F. Brooks, Peter J. Scott, Benjamin L. Viglianti

**TO THE EDITOR:** In the published VISION trial results on ^177^Lu-vipivotide tetraxetan (Pluvicto; Novartis) use in metastatic castration-resistant prostate cancer, the mass and molar activity of prostate-specific membrane antigen (PSMA-617) are not included in the supplemental protocol or in the Food and Drug Administration–approved package insert (1*,*^2^). The radioactivity assay is included as a dose (1,000 MBq/mL) in lieu of a PSMA-617 concentration. For ^177^Lu-DOTATATE (Lutathera; Novartis) and its corresponding trial NETTER-1, the radioactivity assay (370 MBq/mL), the total mass content (X-DOTA^0^-Tyr^3^-octreotate, 10 μg/mL), and the molar activity (53 GBq/μmol) were provided (3). Additionally, the approved PET diagnostic counterparts, ^68^Ga-gozetotide (Locametz; Novartis)/^68^Ga-gozetotide (Illuccix; Telix) and ^68^Ga-DOTATATE (NetSpot; Novartis), include the PSMA-11 and DOTATATE masses (25 and 40 μg, respectively) as part of their package inserts. The injected mass of PSMA-617 in Pluvicto doses is required to calculate specific and molar activity, which is potentially critical in the area of optimizing therapy dosage and frequency in an effort to individualize treatment for maximal treatment efficacy. In patients with poor uptake, excessive cold mass is an important consideration to exclude as a cause for the lack of therapeutic effect.

With no reported PSMA-617 mass in clinical Pluvicto doses, and literature-reported PSMA-617 masses ranging from 125 to 160 μg, accurate measurement of PSMA-617 mass is required (4*,*^5^). Due to patient factors at our therapy clinic, 8 unused clinical Pluvicto doses were available for use in our quality control program. The 8 samples were analyzed using radio–high-performance liquid chromatography with a gradient and isocratic method, at 2 different wavelengths (maximum absorbance intensity, 225 nm; wavelength of maximum absorption, 277 nm). A standard curve was generated for each method and wavelength, and the resulting 8 samples were interpolated from the standard curves (Supplemental Table 1; Supplemental Fig. 1 [supplemental materials are available at http://jnm.snmjournals.org]). Using the 8 clinical Pluvicto doses, we report an average PSMA-617 mass of 6.31 ± 0.72 μg/mL (*n* = 32, 4 samples per Pluvicto vial), which corresponds to a molar activity range of 100–212 GBq/μmol at calibration, depending on the volume of Pluvicto injected (ranging from 7.5 to 12.5 mL, according to the package insert).

We encourage the supplier to provide the community with the total mass content and molar activity, in line with practices for reporting other diagnostic and radiotherapeutic agents in nuclear medicine as is provided with Lutathera. This will alleviate concerns that mass could cause low uptake in select patients and be used for research or clinical trial development centered around receptor expression, occupancy, or turnover. We also encourage the Food and Drug Administration to require the package insert to list the total mass content and molar activity for future approved radiotherapies as the field moves toward planning individual radiotherapy as part of personalized medicine for patients with specialized cases.

## DISCLOSURE

The authors were supported by funding from NIH (R01EB021155; principal investigator, Peter Scott) and the Neuroendocrine Tumor Foundation 2023 NETRF Investigator Award (award 1159892; principal investigator, Benjamin Viglianti). No other potential conflict of interest relevant to this article was reported.

## Notes

**Ivan E. Wang, Allen F. Brooks, Peter J. Scott, Benjamin L. Viglianti***

**University of Michigan Medical Center Ann Arbor, Michigan*

*E-mail: bviglia@med.umich.edu*
